# Receptive and expressive English language assessments used for young children: a scoping review protocol

**DOI:** 10.1186/s13643-017-0471-1

**Published:** 2017-04-04

**Authors:** Laureen J. McIntyre, Laurie-ann M. Hellsten, Julia Bidonde, Catherine Boden, Carolyn Doi

**Affiliations:** 1 Department of Educational Psychology and Special Education, University of Saskatchewan, 28 Campus Drive, Saskatoon, SK S7N 0X1 Canada; 2 Norwegian Institute of Public Health, Nydalen, PO Box 4404, 0403 Oslo, Norway; 3 Leslie and Irene Dube Health Sciences Library, University of Saskatchewan, Room 1400, Academic Health Sciences Building, 104 Clinic Place, Saskatoon, SK S7N 2Z4 Canada; 4 Education and Music Library, University of Saskatchewan, Room 2003, Education Building 28 Campus Drive, Saskatoon, SK S7N 0X1 Canada

**Keywords:** Receptive language, Expressive language, Language assessment, English, Children, Scoping review

## Abstract

**Background:**

The majority of a child’s language development occurs in the first 5 years of life when brain development is most rapid. There are significant long-term benefits to supporting all children’s language and literacy development such as maximizing their developmental potential (i.e., cognitive, linguistic, social-emotional), when children are experiencing a critical period of development (i.e., early childhood to 9 years of age). A variety of people play a significant role in supporting children’s language development, including parents, guardians, family members, educators, and/or speech-language pathologists. Speech-language pathologists and educators are the professionals who predominantly support children’s language development in order for them to become effective communicators and lay the foundation for later developing literacy skills (i.e., reading and writing skills). Therefore, these professionals need formal and informal assessments that provide them information on a child’s understanding and/or use of the increasingly complex aspects of language in order to identify and support the receptive and expressive language learning needs of diverse children during their early learning experiences (i.e., aged 1.5 to 9 years). However, evidence on what methods and tools are being used is lacking.

**Methods:**

The authors will carry out a scoping review of the literature to identify studies and map the receptive and expressive English language assessment methods and tools that have been published and used since 1980. Arksey and O’Malley’s (2005) six-stage approach to conducting a scoping review was drawn upon to design the protocol for this investigation: (1) identifying the research question; (2) identifying relevant studies; (3) study selection; (4) charting the data; (5) collating, summarizing, and reporting the results; and (6) consultation.

**Discussion:**

This information will help these professionals identify and select appropriate assessment methods or tools that can be used to support development and/or identify areas of delay or difficulty and plan, implement, and monitor the progress of interventions supporting the development of receptive and expressive language skills in individuals with diverse language needs (e.g., typically developing children, children with language delays and disorders, children learning English as a second or additional language, Indigenous children who may be speaking dialects of English). Researchers plan to evaluate the effectiveness of the assessment methods or tools identified in the scoping review as an extension of this study.

**Electronic supplementary material:**

The online version of this article (doi:10.1186/s13643-017-0471-1) contains supplementary material, which is available to authorized users.

## Background

Oral language involves speaking and listening in order to communicate [1–3]. Modes of communication are generally described “as either receptive language, which involves receiving and decoding or interpreting language, or expressive language, which is the encoding or production of a message” [4]. Language can be further subdivided into the domains of language form (syntax, morphology, phonology), content (semantics), and use (pragmatics) [1, 4, 5]. Language has been defined as “a code whereby ideas about the world are represented through a conventional system of arbitrary signals for communication” [1]. That is:

Language consists of some aspect of content or meaning that is coded or represented by linguistic form for some purpose or use in a particular context. This three-dimensional view of language is basic to describing the development of language and for understanding language disorders [1].

School-aged children need to actively use language across the curriculum to construct meaning for themselves [2, 6]. Learning language is a primary developmental process for children. It is necessary for children to develop their receptive (ability to understand) and expressive (ability to use) language skills to become effective communicators [2, 7]. A variety of people play a significant role in supporting this process, including parents/guardians, family members, educators, and/or speech-language pathologists [5, 8]. It is expected that younger children’s families and school-aged children’s families and classroom teachers facilitate language development since this primary process also lays the foundation for the learning of a secondary process, written language [2, 9]. Therefore, professionals working to support children’s language development, which include educators and speech-language pathologists, need to assess and monitor children’s receptive and expressive language learning progress for them to become effective communicators and lay the foundation for later developing literacy skills (i.e., reading and writing skills) [7].

Typically, the bulk of a child’s language development occurs in the first 5 years of life when brain development is most rapid [8]. In Canada and the USA, if parents/families are concerned that a child has not met developmental milestones and is not understanding and actively using language to communicate by 18 months of age (i.e., 1.5 years), it is recommended that a formal speech and language assessment be conducted by a speech-language pathologist [10]. There are significant long-term benefits to supporting children’s language and literacy development, such as preventing the loss of developmental potential (i.e., cognitive, linguistic, social-emotional) in early childhood or birth to 9 years of age when children are experiencing a critical period of development [11–13]. Both informal and formal assessment methods and/or tools can be used to evaluate the language skills of children with diverse language needs (e.g., typically developing children, children experiencing language delays or disorders, children learning English as a second or additional language, Indigenous children who may be speaking dialects of English). Informal assessment methods can be defined as assessment methods considering children’s performance or skills that can be easily worked into learning activities such as curriculum-based or dynamic assessments in a classroom [14]. Formal assessment methods can be defined as assessment methods with established expectations for administration, scoring, and interpretation such as standardized tests [14]. Therefore, in order to identify and support the receptive and expressive language learning needs of diverse children aged 1.5 to 9 years, educators and speech-language pathologists need assessments that provide them information on a child’s understanding and/or use of the increasingly complex aspects of language. However, evidence on what tools are being used is lacking. Although one ongoing PROSPERO registered systematic review is considering early language assessment methods [15], this study is markedly different from this scoping review study in three ways. One, the Lyall (2014) review is only including “research trials with 100 or more participants” [15]. This scoping review will consider research studies with varying numbers of participants (i.e., single subject case studies to large-scale longitudinal studies with hundreds of participants, etc.). Two, the Lyall (2014) review is only considering “impaired language development in early childhood. This is an impairment in speech and language that is present in isolation to any other development delay, condition or disease” [15]. This scoping review differs since it considers informal and formal assessment methods or tools being used that can be used to support development and/or identify areas of delay or difficulty and plan, implement, and monitor the progress of interventions supporting the development of receptive and expressive language skills in individuals with diverse language needs (e.g., typically developing children, children with language delays and disorders, children learning English as a second or additional language, Indigenous children who may be speaking dialects of English). Three, the Lyall (2014) review is including studies related to “Preschool children (aged 2–6) only samples of 100 or more; Exclusion: Disease and disorders affecting brain development and language development” [15]. This scoping review will consider a wider age range (i.e., 1.5 to 9 years of age) and does not exclude studies in which subjects have been diagnosed with diseases or disorders that affect brain and language development. The language assessments being used with these individuals need to be considered since their language skills will need to be supported by family members, educators, and speech-language pathologists. Four, we are using a different method of examining the literature. Lyall (2014) is a systematic review, while we are conducting a scoping review. We therefore will carry out a scoping review of the literature to identify studies assessing the receptive and expressive language skills using a variety of assessment methods or tools in this population. Researchers plan to evaluate the effectiveness of the assessment methods or tools identified in the scoping review as an extension of this study.

## Methods/design

We chose a scoping review as the best method to understand and map the receptive and expressive language assessment methods and tools published and used since 1980. Scoping review methodology is particularly useful for examining the breath of the research in a specific topic area. Also, scoping reviews are useful to comprehensively and systematically map the literature and identify key evidence or research gaps. Nonetheless, this type of review is rigorous and methodical in its approach to examining the extent, range, and nature of research activity in a particular field while encompassing both empirical and conceptual research with openly framed questions [16–18]. This study has not been registered in PROSPERO, since scoping reviews are not eligible for inclusion. In designing the protocol for this scoping review, we drew upon Arksey and O’Malley’s (2005) seminal work as well as recent publications [16–19]. Arksey and O’Malley’s (2005) scoping review framework outlines a six-stage approach with each stage discussed below [17]. Adaptations were driven by an intention to develop a feasible approach for reviewing a potential vast body of literature.

### Stage 1: identifying the research questions

Following the recommendations of Arksey and O’Malley (2005), we will follow an iterative process for developing the research question(s) [17]. We will continue doing this as we become increasingly familiar with the literature. A first run trial search informed question development by clarifying the meaning of key terms such as speech (i.e., “verbal means of communicating” ) versus language and formal (e.g., standardized tests) versus informal assessments or methods (i.e., curriculum or dynamic assessments) and identifying key terms that are associated with language but will not have related research included in this review unless language-based tasks are also being assessed (e.g., multi-component or composite assessment tools related to cognition, achievement, or written language) [20]. Our intention to comprehensively examine expressive and receptive English language assessment literature prompted us to develop the following initial questions:

- What is known from the literature about receptive and/or expressive English language assessment methods or tools for children ages 1.5 to 9 years?
- What are the patterns of assessment use? For example, what is the purpose of assessment, in what types of settings have the assessment been used, what are the characteristics or properties of the assessment, who is using the assessment (i.e., personnel/staff/parent assessing the child), and who is being evaluated using the assessment (client, student, etc.)?

### Stage 2: identifying relevant studies

The aim of the scoping review will be to comprehensively address broad research questions; however, parameters are required to guide the search strategy. At this stage, the team decided upon the criteria for eligibility, the databases that would be searched, formulated a search strategy, and identified key terms.

#### Eligibility criteria

The following inclusionary criteria will be used to guide the search and will also be used when screening or reviewing the articles/publications (see PICO-T summary in Table 1):

- Published in any language. Members of the research team have the ability to translate select languages (e.g., Spanish, Portuguese). Any languages not able to be translated by team members will be carried out by a professional translator.
- 1980 to present.
- Children 1.5 to 9 years (typically and atypically developing) which includes children with an exceptionality(ies) or learners, ranging from those with severe disabilities to those who demonstrate gifts and/or talents, who require special education if they are to reach their full human potential [21], children learning English as an additional or second language, and children using dialects of English.
- Publications that target children of any gender or ethnicity in any setting (school, private practice, hospital, home, etc.) and evaluate the child using an English language receptive and/or expressive assessment tool, a translation of a receptive and/or expressive tool conducted in English, or a bilingual/multilingual assessment tool that considers English as one of the languages being assessed.
- Receptive and/or expressive language assessments that provide information about the child’s understanding and/or use of the increasingly complex aspects of one or more of the five areas of language being tested (i.e., phonology, morphology, syntax, semantics, and pragmatics). This includes assessments considering pre-verbal (e.g., vocalizations, eye gaze) and/or nonverbal communication (e.g., gestures, facial expressions, body positions) and nonverbal languages (e.g., finger spelling assessments).
- Publications including formal or standardized and informal or non-standardized tests, questionnaires, checklists, scales, protocols, inventories, and language sampling.
- We will include assessments/tests that have been *published* in the literature (that means peer reviewed journal articles of primary and secondary research, books or book chapters, grey literature including dissertations, or guidelines).
- Multicomponent or composite assessment tools will be included if there is a receptive and/or expressive language component or subscale (e.g., cognitive, achievement, or written language assessments that also consider receptive and/or expressive language skills).
- Completed systematic reviews. In the event a systematic review has been completed that is relevant to the content of this review before researchers enter stage 5 (collating, summarizing, and reporting the results), it will be added to the results.

**Table 1:** PICO-T framework: inclusionary criteria

Population	Children of any gender or ethnicity, 1.5 to 9 years, typically and atypically developing
Intervention	Receptive and/or expressive language assessments*, in English or a translation of a language assessment conducted in English, or a bilingual/multilingual assessment tool that considers English as one of the languages being assessed *Assessments in one or more of the five areas of language (i.e., phonology, morphology, syntax, semantics, and/or pragmatics) *Pre-verbal and/or nonverbal communication and nonverbal languages (i.e., finger spelling assessments) *Multicomponent or composite assessment tool with a receptive and/or expressive language component or subscale (e.g., cognitive, achievement, or written language assessment)
Comparison	Unrestricted (either with or without comparator)
Outcomes	Map the receptive and/or expressive English language assessments (formal or standardized and informal or non-standardized tests, questionnaires, checklists, scales, protocols, inventories, and language sampling) that have been published and used
Time frame	Articles/publications from 1980 to present

* denotes specific types of English receptive and/or expressive language assessments that will be included

The following exclusionary criteria will be used to guide the search and will also be used when screening or reviewing the articles/publications. Articles/publications will be excluded if the focus is related to:

- Aspects of written language (e.g., spelling, literary devices such as irony).
- Speech reception/perception, analysis, classification (i.e., phonetics or study/classification of speech sounds), or production (e.g., intelligibility, articulation, mechanical aspects of communication).
- Auditory perception, detection, discrimination, processing, or signaling software testing.
- Cognitive skill development or disorders (e.g., attention, memory, problem solving, executive functioning, thinking, reasoning).
- Analysis or approaches for tool development.
- Review of industry websites or catalogues.
- Systematic review or study protocols.
- Assessing a person’s potential for learning a language.
- Assessments that are only focused on the development of a language other than English (e.g., French, Spanish).

If books and/or chapters are found that contain the same information presented in an article, the article will be used and the books/chapters will be excluded from the search.

#### Databases

Core subject databases have been selected in education (ERIC Ovid, CBCA Education, Education Database), psychology (PsycTests, Mental Measurements Yearbook with Tests in Print, PsycInfo), health sciences (Medline, CINAHL), and linguistics (Linguistics and Language Behavior Abstracts or LLBA). Core multidisciplinary databases covering these topics have also been selected (ProQuest Dissertations and Theses Global). In addition, we will search the Health Technology Assessment portal (HTA database).

#### Search strategy

Two librarians who are part of the research team will be instrumental in choosing and applying search terms to comply with several databases in the health and social sciences, in consultation with the subject experts on the research team. They will develop and test a search strategy; based on their expertise and the outline of this project, they will select keywords, controlled vocabulary terms, and filters to maximize sensitivity and specificity within the search. An interactive process of trial searches in one key database (OVID Medline), subject expert feedback, and verification of search results in reference to a sample set of key articles will continue until no further refinements are identified. The search strategy from OVID Medline will then be optimized for the other databases. Any new terms identified in the optimization stage will be incorporated into the strategies in all databases. A search strategy will be developed for each database by one of the librarians and subsequently reviewed by the other using the PRESS Peer Review of Electronic Search Strategies checklist [22].

To supplement the scholarly literature, a search for grey literature will be developed in accordance with the procedures recommended by the Canadian Agency for Drugs and Technologies in Health (CADTH) [23]. The grey literature search will be limited to eight English speaking national and international speech-language pathology professional organizations and association websites: Speech-Language and Audiology Canada, American Speech-Language-Hearing Association, Speech Pathology Australia, The Irish Association of Speech and Language Therapists, New Zealand Speech Language Therapists Association, Association of Speech and Language Therapists in Independent Practice (UK), The Royal College of Speech and Language Therapists UK, and the International Association of Logopedics and Phoniatrics. The aim will be to detect publically accessible policy papers, white papers, reports, or other publications about language assessments employed by practicing professionals that might not be found in the scholarly literature.

The complete and final search strategy will be provided in a follow-up publication in accordance with PRISMA guidelines and recommendations by Rader et al. (2014) [24]. Although developed for systematic reviews, no similar guidelines exist specifically for scoping reviews and the search methods are sufficiently similar that we feel this is appropriate. Further search strategy details across bibliographic databases will be available from the first author. Upon completion, the searches from each database will be documented and references will be imported into a bibliographic software for duplicate elimination. De-duplicated references will be imported into the web application Rayyan for screening [25].

### Stage 3: study selection

Two reviewers will independently screen citations for inclusion. Each pair of reviewers will have a content expert. At title and abstract level, uncertainties from the reviewers will not automatically eliminate the record. We will determine final inclusion at second level (full-text screening). A third reviewer will arbitrate in cases there is disagreement at any stage until consensus is reached. Our team content expert will be part of all consensus meetings. The full text of all papers identified as having potential for inclusion will be requested. One author will abstract data from papers included, and another will check the data using a priori checklists. A PRISMA flow diagram will report final numbers once the scoping review is completed [26].

### Stage 4: charting the data

We will collect and sort key pieces of information from the selected full-text articles. We will use standardized forms created for this purpose. We will train and examine the charting consistency with the questions and purpose. Additional categories may emerge during the data collection process; in which case, in consultation with the team, we will adapt and restructure the forms. Extracted data will include:

- General information/study characteristics. For example, authors’ country of publication, year of publication, purpose of the tool, language of publication, setting (school, hospital, home).
- Characteristics of the children included, such as age, children’s first language, female/male ratio, inclusion and exclusion criteria, education level.
- Tool characteristics. For example if the tool has a single purpose or multiple purposes (i.e., composite assessment), is commercially available, is a sub-test in a larger tool, is subjectively or objectively measured, or if it has any challenges and limitations.
- Assessors’ qualifications (i.e., is there a required level of training needed to administer the tool).

We will also make note of any provided information related to the psychometric properties, or efficacy, of the assessments (e.g., reliability or validity evidence).

### Stage 5: collating, summarizing, and reporting the results

The unique purpose of a scoping review is to aggregate the findings and present an overview rather than a meta-synthesis reporting results on narrowly defined questions.

We plan to:

- Map results (i.e., main sources and types of evidence available) from the literature.
- Provide a descriptive summary.

Extracted data from all included articles will be summarized to describe the assessment methods or tools used with children. Since this is a scoping review, there is no principal summary measure. The following analyses will be completed:

1. The number of times a given measure was used will be counted and expressed as a percentage of the total number of times a language measure tool was used in the included articles.
2. The number of times a given study purpose was reported will be counted. For example, possible study purposes may include investigating the relation between language assessment and another variable or examining the effects of a particular intervention.
3. For each receptive and/or expressive language measure identified, principle characteristics such as language and country of origin or information concerning its validity, reliability, responsiveness, and/or interpretability (e.g., interpretation of scores relative to normative data, cut-off values) will be gathered.

Additionally we will be able to identify gaps in the literature and action areas and determine where more in-depth analysis is required. We will follow PRISMA reporting guidelines for systematic reviews to accurately report the results and analysis summary [26].

### Stage 6: consultation

This stage is optional in the foundational framework by Arksey and O’Malley (2005) [17]. We have chosen to undertake this step at protocol and review stages to obtain feedback from relevant stakeholders inviting feedback on our initial questions and methodological decisions and comments on our preliminary results. This will be important to ensure high quality and relevant results are derived to the target audience (e.g., users of these types of tools/assessments). Thus, clinicians knowledgeable of this study’s content and methodologists will be selected from the areas of speech-language pathology (a Canadian and/or American certified speech-language pathologist) and evidence synthesis. We will include their feedback to our work at the protocol and during the review process.

We have included our PRISMA-P 2015 checklist as an Additional file 1. Knowledge mobilization for this research will include professional and scholarly dissemination (i.e., peer-reviewed manuscript and conference submissions).

## Discussion

Formal and informal language assessment methods or tools are being used and discussed by educators and speech-language pathologists in the research and professional literature. It is important to support these professionals in identifying and selecting appropriate assessment methods or tools that can be used to identify areas of delay or difficulty and plan, implement, and monitor the progress of interventions supporting the development of receptive and expressive language skills in individuals with diverse language needs (e.g., typically developing children, children with language delays and disorders, children learning English as a second or additional language, Indigenous children who may be speaking dialects of English). We presented our protocol for systematically conducting a scoping review of published literature specific to receptive and expressive language assessment tools used for children aged 1.5 to 9 years. This scoping review offers a feasible means for synthesizing unknown research literature specific to receptive and expressive language assessment methods or tools. As this will be a first scoping review within this topic area, our results will advance the knowledge in the field. Results will provide unique insights concerning the extent and scope of receptive and expressive language assessment methods or tools used for children aged 1.5 to 9 years for research and clinical practice. We will identify research trends and potential gaps specific to our research questions. A reflective analysis of this large corpus of literature as a whole may reveal new research directions for receptive and expressive language assessment tools used for children aged 1.5 to 9 years.

## Additional file

Additional file 1: PRISMA-P 2015 checklist. (DOCX 29 kb)
